# Cross-cultural differences in exercise motivation among U.S. and Korean college students: evidence from the Exercise Motivations Inventory-2 (EMI-2)

**DOI:** 10.3389/fpubh.2026.1831142

**Published:** 2026-06-26

**Authors:** Dongwook Cho

**Affiliations:** College of Physical Education, Keimyung University, Daegu, Republic of Korea

**Keywords:** college students, cross-cultural comparison, cultural values, Exercise Motivation Inventory-2 (EMI-2), health promotion, motivation for exercise, physical activity

## Abstract

**Background:**

As college students increasingly face academic and social pressures, maintaining regular physical activity has become an important public health concern. However, Preliminary data indicate that physical inactivity remains a global public health concern, with college students experiencing barriers influenced by social and cultural factors. Thus, this study aimed to examine cross-cultural differences in exercise motivation among college students in the United States and South Korea.

**Methods:**

Participants were recruited from two public 4-year universities located in the southern United States and from two 4-year universities in Korea. A total of 752 college students (373 U.S. and 379 Korean) were analyzed using the Exercise Motivation Inventory-2 (EMI-2).

**Results:**

The findings indicated that U.S. students generally exhibit higher motivation for exercise, particularly that inspired by stress management (*p* < 0.001), revitalization (*p* < 0.001), enjoyment (*p* < 0.001), and health-related factors, while Korean students prioritize social recognition (*p* = 0.007). Additionally, U.S. female students demonstrated greater motivation across all subscales than their Korean counterparts, whereas Korean male students emphasized challenge (*p* = 0.027) and social recognition (*p* < 0.001) more than U.S. males.

**Conclusion:**

The present findings indicate that exercise motivation differs between U.S. and Korean college students across several motive dimensions. The results suggest that some exercise motives may be relatively stable across student populations, whereas others may be more sensitive to educational and social context. These findings may inform future efforts to design context-sensitive physical activity promotion strategies in university settings.

## 1 Introduction

The 2022 World Health Organization (WHO) Global Status Reports on Physical Activity estimated that more than 23% of adults and 81% of adolescents do not meet regular exercise levels (1). More than 1.4 billion people in the world’s adult population are insufficiently active, and there has been no improvement in global levels of physical activity since 2001 (1). Approximately 3.2 million adults’ deaths are directly attributed to lack of exercise or physical inactivity. Other studies indicate that 54.4% of adults in Korea (2) and 31% of adults in the U.S. (3) do not follow recommended levels of physical activity. More specifically, physical activity among individuals in their early 20s, including college students, decreased by 3% in the U.S. (1, 4) and 5% in Korea (2, 5) compared to 2010.

Regular physical activity or exercise is essential for promoting overall health and well-being, but many individuals, particularly college students, struggle to engage in sufficient exercise (6, 7). The college years represent a critical period in the transition to adulthood, characterized by numerous academic, social, and lifestyle changes. University students frequently manage academic workload, time pressure, and personal responsibilities at the same time, leaving limited room for regular physical activity. Previous studies have nevertheless shown that physical activity is associated with several beneficial outcomes, including better psychological well-being, lower anxiety and depressive symptoms, and improved health-related quality of life (3). Accordingly, the overall prevalence of regular physical activity has diminished among college students in both Korea and the United States, as individuals increasingly encounter interpersonal barriers and structural limitations that restrict participation in exercise (8).

The importance of exercise participation among college students has risen rapidly as the target audience for exercise motivation research (9). Understanding the factors that motivate individuals to engage in exercise is crucial for designing effective interventions to promote physical activity among college students. Motivation serves as the driving force behind behavior, influencing individuals’ decisions to initiate and sustain participation in physical activity (10–12). Self-Determination Theory has been widely applied to exercise behavior research because it offers a useful framework for understanding how motivational orientation relates to participation patterns. In general, motives based on enjoyment, challenge, and personal value are more closely associated with regular physical activity, while externally regulated motives tend to be less strongly connected to sustained exercise participation (13, 14). Exercise motivation is also multifaceted, encompassing diverse psychological, social, and cultural factors. Intrinsic motivations for exercise stem from individuals’ internal desires and preferences, such as the enjoyment of physical activity, the sense of accomplishment from mastering new skills, and the intrinsic satisfaction of movement and exercise (15). Recent studies have continued to support Self-Determination Theory as a useful framework for understanding exercise behavior among university students. Motives reflecting enjoyment, personal value, and intrinsic satisfaction are generally associated with higher levels of physical activity participation and better psychological well-being, whereas motives based mainly on external demands or social approval show weaker and less consistent associations with sustained exercise engagement (13, 14, 16). Accordingly, examining exercise motivation across multiple dimensions is essential when studying physical activity among college students.

Exercise motivation among college students is a global interest, as young adults often grapple with incorporating regular physical activity into busy academic schedules (4, 9). Understanding differences and similarities in exercise motivation between U.S. and Korean college students provides valuable insights into cultural and societal factors influencing this demographic’s fitness behavior (13, 17). The previous studies demonstrated the relevance of Self-Determination Theory for understanding exercise motivation among university students, showing that different motivational orientations are associated with physical activity participation and mental health outcomes (13, 14). In addition, social factors such as peer relationships and perceived social support have been identified as important correlates of exercise intentions and adherence among college students (18).

Comparing U.S. and Korean college students is theoretically meaningful because it allows examination of how exercise motives are organized within two educational and social contexts that differ in campus recreation opportunities, participation norms, and broader expectations surrounding physical activity (13, 19–22). Rather than treating the two countries simply as separate national samples, this comparison helps clarify whether particular motives are broadly shared across settings or more sensitive to contextual conditions. In this way, the study contributes to a more refined understanding of how cultural and institutional environments may shape the relative salience of exercise motives among college students.

The United States and South Korea were chosen for comparison because previous research has examined exercise participation among university students from different perspectives in each country. Studies conducted in the United States have often focused on health-related motives, recreational sport participation, and campus-based physical activity opportunities (18), whereas research in South Korea has emphasized exercise enjoyment, adherence, and barriers to participation among university students (23, 24). These findings suggest that the context in which exercise participation occurs may differ between the two countries, providing a rationale for comparing exercise motivation across student populations. In addition, academic pressure, peer expectations, and time constraints have been identified as important factors related to physical activity patterns among Korean college students (25, 26). Although increasing attention has recently been directed toward the mental and physical health benefits of exercise among Korean youth (23, 24, 27), differences in social expectations and participation contexts may contribute to distinct motivational profiles. Examining exercise motivation in these two national contexts may therefore provide valuable insights into how exercise motives vary among college students.

This study examined exercise motivation among college students in the United States and South Korea. Although exercise motivation has been widely studied in university settings, few studies have compared motivational profiles between students from these two countries using the same measurement instrument. Previous research has shown that exercise participation is associated with social, environmental, and motivational factors among university students (12, 19, 22). Comparing students from the United States and South Korea may help clarify whether exercise motives differ across national contexts and contribute to a broader understanding of exercise motivation in young adults.

Exercise motivation has practical relevance because participation in physical activity often changes during the university years. Understanding which motives are more strongly endorsed by different student groups may help researchers and practitioners better understand factors associated with exercise participation. In addition, comparing students from the United States and South Korea provides an opportunity to examine exercise motivation in two different university settings using the same measurement instrument. The findings may add to the existing evidence on exercise motivation among young adults and provide a basis for future comparative research involving university student populations. Therefore, the primary purpose of this study was to compare exercise motivation between U.S. and Korean college students using the Exercise Motivations Inventory-2 (EMI-2). In addition, secondary subgroup analyses were conducted to examine whether motivational patterns differed between male and female students within each country. The study tested the following hypotheses:

*Hypothesis 1 (H1)*: Exercise motivation would differ between U.S. and Korean college students.

*Hypothesis 2 (H2) and Hypothesis 3 (H3)*: Secondary subgroup analyses would examine whether exercise motivation differed between U.S. and Korean female and college students, respectively.

These hypotheses are grounded in prior research showing that social context and environmental conditions may be associated with differences in motivation and physical activity participation (17, 20). From the perspective of Self-Determination Theory, such differences may be reflected in the relative salience of motives linked to enjoyment, personal health, stress management, social recognition, and group-based participation (13, 14). However, relatively little is known about how these motivational patterns are organized across national university settings. The comparison between U.S. and Korean college students is therefore theoretically informative because these contexts differ in social, educational, and cultural environments that may shape the relative importance of exercise motives in different ways. This cross-national design offers an opportunity to examine whether some motives are broadly shared across settings, whereas others may be more closely tied to local norms and participation environments. In this sense, the present study contributes not only to the description of between-group differences, but also to a broader understanding of how exercise motivation may be structured across distinct social contexts. Accordingly, the present study examined differences in exercise motivation between college students in the United States and South Korea.

## 2 Methods

### 2.1 Participants

This study used convenience sampling for an exploratory comparison of U.S. and Korean college students. U.S. college student participants were recruited from two public 4-year universities located in the southern United States, while Korean college students came from two 4-year universities in the regions of Chungbuk and Seoul that each had approximately 25,000 students. The participants were informed of the research’s anonymity and that proper approval had been obtained prior to taking the survey.

Data were collected between September 2024 and February 2025 through convenience sampling. Students enrolled in undergraduate courses were invited via classroom announcements and institutional email distribution lists. A total of 400 Korean college students completed the study questionnaire; 21 responses were removed due to being incomplete, leaving a total of 379 (185 males and 194 females). For the U.S. sample, 400 questionnaires were collected (200 online and 200 paper-based). After excluding 27 incomplete responses, 373 questionnaires (184 males and 189 females) were retained for analysis. Participants’ ages were limited to between 18 and 25 years old, with an average age of 20.9 ± 1.7 years for Korean students and 21.2 ± 1.6 years for U.S. students to better represent typical college-aged populations.

### 2.2 Procedures and ethical considerations

The research protocol was designed to ensure methodological transparency and adherence to ethical standards, enabling replication across international settings. Data collection took place at two public universities in the United States and two universities in South Korea. The study protocol was reviewed and approved by the Institutional Review Boards (IRBs) of the Alcorn State University (IRB# 030217-002). All participants provided informed consent before participation. They were informed of their right to withdraw at any time without penalty. Confidentiality of all data was maintained in accordance with ethical standards of research. The study adhered to the principles outlined in the Declaration of Helsinki for research involving human subjects.

Surveys were distributed in-person and online, depending on local context and participant preference, with standardized instructions provided by trained facilitators in both countries. All responses were anonymized and securely stored, and data management practices reflected open science principles with detailed documentation of procedures, instruments, and analysis workflows.

### 2.3 Instruments

This study utilized the Exercise Motivation Inventory-2 (EMI-2), developed by Markland and Ingledew, comprising 51 items rated on a six-point Likert scale from 1 (not at all true for me) to 6 (very true for me) (28). The instrument consists of 14 subscales: affiliation, appearance, challenge, competition, enjoyment, health pressure, ill-health avoidance, nimbleness, positive health, revitalization, social recognition, strength and endurance, stress management, and weight management (see Table 1). The EMI-2 assesses exercise motivation across both intrinsic and extrinsic dimensions, including health, psychological, social, appearance, performance, and enjoyment motives. Scores for each subscale were calculated by averaging 3 to 4 relevant items, based on the EMI-2 scoring guidelines (29, 30). The Korean version of EMI-2 had previously been translated and validated in Korean college students using standard forward-translation and back-translation procedures, with evidence of adequate reliability and validity. In the present study, measurement invariance analyses further indicated that the English and Korean versions were equivalent across groups (24).

**Table 1:** Exercise Motivation Inventory-2 (EMI-2) subscales, sample items, numbers of items, and Cronbach’s alpha coefficient.

Subscale	Sample item	No. of items	*α*
Affiliation	To spend time with friends	4	0.857
Appearance	To have a good body	4	0.915
Challenge	To give me goals to work toward	4	0.798
Competition	Because I like trying to win in physical activities	4	0.911
Enjoyment	Because I feel at my best when exercising	4	0.873
Health pressures	Because my doctor advised me to exercise	3	0.886
Ill-health avoidance	To prevent health problems	3	0.795
Nimbleness	To maintain flexibility	3	0.833
Positive health	Because I want to maintain good health	3	0.737
Revitalization	To recharge my batteries	3	0.921
Social recognition	To show my worth to others	4	0.842
Strength and endurance	To develop my muscles	4	0.805
Stress management	To give me space to think	4	0.905
Weight management	To stay slim	4	0.877

### 2.4 Data analysis

Data were analyzed using IBM SPSS Statistics 27 to measure the data reliability; the demographic characteristics of the participants, and the descriptive statistics of EMI-2. SPSS Amos 27 was additionally utilized to verify the measurement invariance across culture and gender. The average scores of the 14 EMI-2 subscales served as the dependent variables. Because the distributions were non-normal and the sample was obtained through convenience sampling, differences between-group were examined using the Mann–Whitney *U* test. Given the large number of comparisons, *p-*values were interpreted together with effect sizes rather than using a Bonferroni correction, which may overly reduce statistical power. In addition to p values, standardized *Z* values and effect sizes were reported to indicate the magnitude of group differences. The effect sizes were derived from the standardized test statistics and were included because statistical significance alone does not adequately reflect the practical importance of the observed differences in nonparametric group comparisons.

The internal consistency of the Exercise Motivation Inventory-2 (EMI-2) was evaluated using Cronbach’s alpha. The overall 51-item scale demonstrated good reliability, with a Cronbach’s alpha coefficient of 0.875. Reliability was also confirmed for each of the 14 subscales of the EMI-2 (see Table 1). Descriptive statistics were computed for the demographic characteristics of the sample, including age, gender, and nationality. Means and standard deviations for each EMI-2 subscale were calculated to summarize exercise motivation profiles across the full sample and across the primary cross-cultural comparison, with secondary subgroup analyses conducted separately for female and male students within each country. The primary cross-cultural comparison was examined using the Mann–Whitney *U* test, and secondary subgroup comparisons were conducted for male and female students within each country.

Before conducting cross-group comparisons, measurement invariance of the Exercise Motivation Inventory-2 (EMI-2) across culture (U.S. vs. Korea) and gender (male vs. female) was examined using multi-group confirmatory factor analysis (CFA). Sequential tests of configural, metric, and scalar invariance were conducted, following standard procedures outlined (30, 31). Model fit was assessed using multiple indices, including the chi-square statistic, Comparative Fit Index (CFI), Tucker–Lewis Index (TLI), Root Mean Square Error of Approximation (RMSEA), and Standardized Root Mean Square Residual (SRMR). In line with commonly accepted guidelines in structural equation modeling, CFI and TLI values of at least 0.90 were interpreted as indicating an acceptable fit, with values approaching 0.95 reflecting a better fit. For RMSEA, values below 0.08 were regarded as acceptable, whereas values below 0.06 suggested a close fit. SRMR values below 0.08 were also considered indicative of an acceptable model fit (32, 33). The results indicated acceptable fit for the configural model (CFI = 0.931, TLI = 0.912, RMSEA = 0.048, SRMR = 0.041), suggesting that the factor structure was similar across groups. Subsequent tests for metric (ΔCFI = 0.004) and scalar (ΔCFI = 0.006) invariance also met the recommended criteria, indicating that the EMI-2 demonstrated measurement equivalence across both culture and gender. Establishing measurement invariance confirms that the EMI-2 measures exercise motivation equivalently across groups, permitting valid mean comparisons and interpretation of observed group differences.

## 3 Results

**Figure 1:**
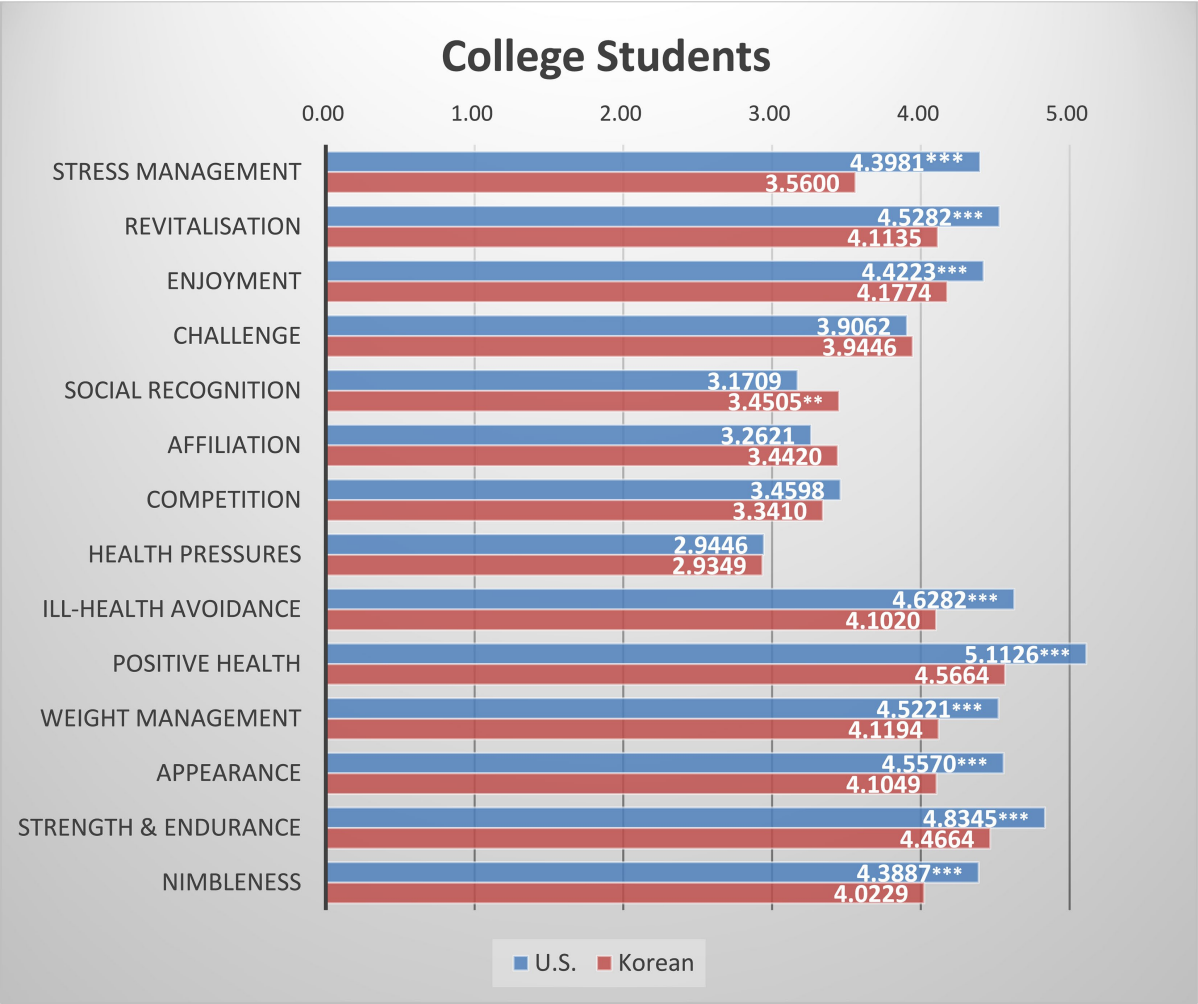
Mean exercise motivation scores for U.S. and Korean college students across the 14 EMI-2 subscales. **p < 0.01, ***p < 0.001.

The comparison between U.S. and Korean college students revealed several significant differences in exercise motivation (see Table 2). U.S. college students reported significantly higher motivation for stress management (*p* < 0.001, Cohen’s *d* = 0.72), revitalization (*p* < 0.001, Cohen’s *d* = 0.38), and enjoyment (*p* < 0.001, Cohen’s *d* = 0.19). They also scored significantly higher on health- and body-related motives, including ill-health avoidance (*p* < 0.001, Cohen’s *d* = 0.52), positive health (*p* < 0.001, Cohen’s *d* = 0.63), weight management (*p* < 0.001, Cohen’s *d* = 0.33), appearance (*p* < 0.001, Cohen’s *d* = 0.44), strength/endurance (*p* < 0.001, Cohen’s *d* = 0.35), and nimbleness (*p* < 0.001, Cohen’s *d* = 0.31). Meanwhile, social recognition was the only exercise motivation for which Korean college students scored significantly higher than U.S. college students in this sample (*p* = 0.007, Cohen’s *d* = −0.22). There were no group differences in EMI-2 scores between U.S. and Korean college students in the subscales of challenge, affiliation, competition, and health pressures as exercise motivations. For better understanding, Figure 1 shows the mean differences in exercise motivation between U.S. and Korean college students as a bar graph. Among the 14 subscales, Korean college students scored significantly higher than U.S. college students only on social recognition. Although Korean students showed descriptively higher means for challenge and affiliation, these differences were not statistically significant. In the case of the health pressure subscale, U.S. and Korean college students were almost identically motivated (see Figure 1).

**Table 2:** Mean differences and ranking of exercise motivations between U.S. and Korean college students.

Subscale	Nationality	*N*	*M*	*SD*	Rank	*Z*	*p*	Cohen’s *d*
Stress management	U.S.	373	4.40	1.24	8	−9.637	<0.001	0.72
	Korea	379	3.56	1.10	10			
Revitalization	U.S.	373	4.53	1.11	5	−5.171	<0.001	0.38
	Korea	379	4.11	1.13	5			
Enjoyment	U.S.	373	4.42	1.30	7	−3.285	<0.001	0.19
	Korea	379	4.18	1.17	3			
Challenge	U.S.	373	3.91	1.36	10	−0.198	0.843	−0.02
	Korea	379	3.94	1.08	9			
Social recognition	U.S.	373	3.17	1.45	13	−2.696	0.007	−0.22
	Korea	379	3.45	1.03	11			
Affiliation	U.S.	373	3.26	1.45	12	−1.760	0.078	−0.13
	Korea	379	3.44	1.26	12			
Competition	U.S.	373	3.46	1.57	11	−1.105	0.269	0.08
	Korea	379	3.34	1.37	13			
Health pressures	U.S.	373	2.94	1.39	14	−0.299	0.765	0.01
	Korea	379	2.93	1.09	14			
Ill-health avoidance	U.S.	373	4.63	1.07	3	−7.073	<0.001	0.52
	Korea	379	4.10	0.97	7			
Positive Health	U.S.	373	5.11	0.86	1	−9.092	<0.001	0.63
	Korea	379	4.57	0.85	1			
Weight management	U.S.	373	4.52	1.25	6	−4.851	<0.001	0.33
	Korea	379	4.12	1.20	4			
Appearance	U.S.	373	4.56	1.09	4	−6.493	<0.001	0.44
	Korea	379	4.10	1.02	6			
Strength and endurance	U.S.	373	4.83	1.09	2	−5.671	<0.001	0.35
	Korea	379	4.47	0.99	2			
Nimbleness	U.S.	373	4.39	1.24	9	−4.407	<0.001	0.31
	Korea	379	4.02	1.18	8			

In addition to examining the mean differences, Table 2 also ranks the overall importance of specific exercise motivations. The ranking analysis should be interpreted as the within-group priority of each motive rather than a comparison of between-group mean differences. In this study, enjoyment had a higher mean score among U.S. students, but it was ranked more highly within the Korean group. Thus, the ranking results show relative importance inside each group, while the mean comparisons in the tables indicate whether the two groups differed statistically. Health pressures were the lowest ranked exercise motivation for both U.S. and Korean college students. Interestingly, enjoyment ranked higher within the Korean group, whereas ill-health avoidance ranked higher within the U.S. group. These rankings reflect within-group priority rather than between-group mean differences.

**Figure 2:**
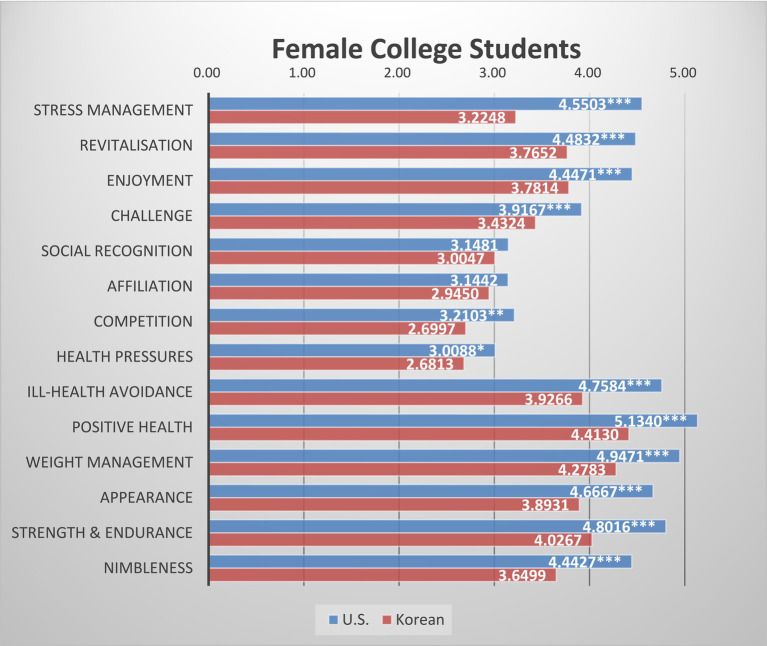
Mean exercise motivation scores for U.S. and Korean female college students across the 14 EMI-2 subscales. *p < 0.05, **p < 0.01, ***p < 0.001.

Turning from the results for college students overall to those for students of a specific gender, Table 3 compares exercise motivation means between U.S. and Korean female college students. The findings of this sample indicated that U.S. female college students had statistically significant higher exercise motivation scores than Korean female college students for stress management (*p* < 0.001, Cohen’s *d* = 1.12), revitalization (*p* < 0.001, Cohen’s *d* = 0.59), enjoyment (*p* < 0.001, Cohen’s *d* = 0.51), challenge (*p* < 0.001, Cohen’s *d* = 0.39), competition (*p* = 0.005, Cohen’s *d* = 0.34), health pressures (*p* = 0.027, Cohen’s *d* = 0.27), ill-health avoidance (*p* < 0.001, Cohen’s *d* = 0.86), positive health (*p* < 0.001, Cohen’s *d* = 0.79), weight management (*p* < 0.001, Cohen’s *d* = 0.60), appearance (*p* < 0.001, Cohen’s *d* = 0.70), strength/endurance (*p* < 0.001, Cohen’s *d* = 0.70), and nimbleness (*p* < 0.001, Cohen’s *d* = 0.64). Although U.S. female students also had higher mean scores for social recognition and affiliation, these differences were descriptive only and did not reach statistical significance. Overall, Figure 2 shows that U.S. female students scored higher than Korean female students across all 14 subscales, but only 12 of these differences were statistically significant.

**Table 3:** Mean differences and ranking of exercise motivations between U.S. and Korean female college students.

Subscale	Nationality	*N*	*M*	*SD*	Rank	*Z*	*p*	Cohen’s *d*
Stress management	U.S.	189	4.55	1.18	6	−9.286	<0.001	1.12
	Korea	194	3.22	1.19	10			
Revitalization	U.S.	189	4.48	1.17	7	−5.486	<0.001	0.59
	Korea	194	3.77	1.22	7			
Enjoyment	U.S.	189	4.45	1.32	8	−4.722	<0.001	0.51
	Korea	194	3.78	1.33	6			
Challenge	U.S.	189	3.92	1.32	10	−3.572	<0.001	0.39
	Korea	194	3.43	1.19	9			
Social recognition	U.S.	189	3.15	1.35	12	−0.965	0.334	0.13
	Korea	194	3.00	0.96	11			
Affiliation	U.S.	189	3.14	1.41	13	−1.208	0.227	0.15
	Korea	194	2.94	1.30	12			
Competition	U.S.	189	3.21	1.61	11	−2.823	0.005	0.34
	Korea	194	2.70	1.35	13			
Health Pressures	U.S.	189	3.01	1.39	14	−2.217	0.027	0.27
	Korea	194	2.68	1.06	14			
Ill-Health avoidance	U.S.	189	4.76	1.00	4	−7.258	<0.001	0.86
	Korea	194	3.93	0.94	4			
Positive health	U.S.	189	5.13	0.86	1	−7.189	<0.001	0.79
	Korea	194	4.41	0.96	1			
Weight management	U.S.	189	4.95	1.09	2	−6.099	<0.001	0.60
	Korea	194	4.28	1.14	2			
Appearance	U.S.	189	4.67	1.15	5	−6.886	<0.001	0.70
	Korea	194	3.89	1.07	5			
Strength and endurance	U.S.	189	4.80	1.10	3	−6.666	<0.001	0.70
	Korea	194	4.03	1.10	3			
Nimbleness	U.S.	189	4.44	1.22	9	−5.773	<0.001	0.64
	Korea	194	3.65	1.25	8			

Even if U.S. female college students had higher exercise motivations, the results of the ranking results of this study indicated no difference among the five highest ranked exercise motivations, namely positive health, weight management, strength/endurance, ill-health avoidance, and appearance. Both groups ranked health pressures lowest among all the exercise motivations. The greatest difference in the two groups’ rankings is that the stress management subscale was the sixth highest motivation for U.S. female college students but only the tenth highest for Koreans.

**Figure 3:**
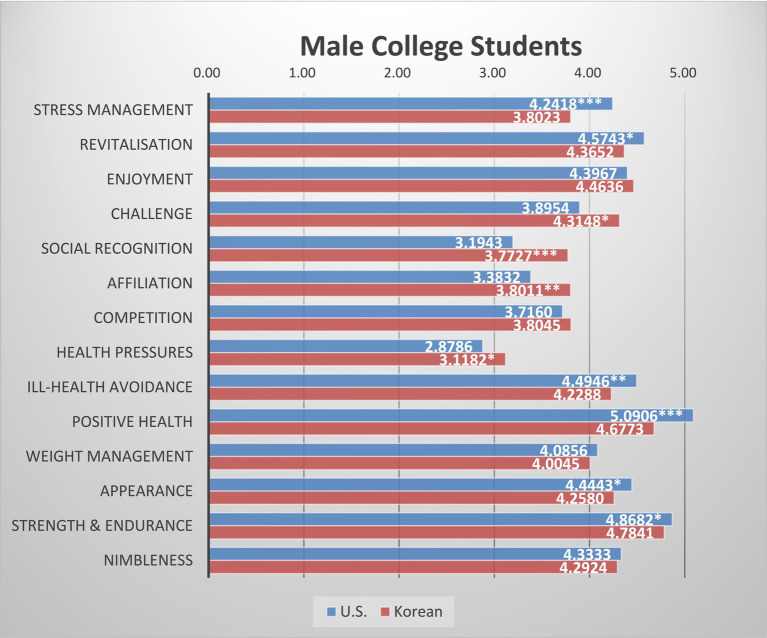
Mean exercise motivation scores for U.S. and Korean college male students across the 14 EMI-2 subscales. *p < 0.05, **p < 0.01, ***p < 0.001.

Table 4 compared exercise motivation among male college students between the U.S. and Korea. The findings revealed that U.S. male students scored significantly higher on the stress management (*p* < 0.001, Cohen’s *d* = 0.39), revitalization (*p* = 0.017, Cohen’s *d* = 0.21), ill-health avoidance (*p* = 0.005, Cohen’s *d* = 0.26), positive health (*p* < 0.001, Cohen’s *d* = 0.53), appearance (*p* = 0.036, Cohen’s *d* = 0.19), and strength/endurance (*p* = 0.042, Cohen’s *d* = 0.09) exercise motivations than Korean male students. On the other hand, the mean scores for the exercise motivation subscales of challenge (*p* = 0.027, Cohen’s *d* = −0.37), social recognition (*p* < 0.001, Cohen’s *d* = −0.45), affiliation (*p* = 0.006, Cohen’s *d* = −0.32), and health pressures (*p* = 0.020, Cohen’s d = −0.19) were significantly higher for Korean male college students. In addition, Figure 3 indicates that U.S. male college students ranked eight exercise motivations relatively higher, while six subscales provided higher motivations for Korean male college students.

**Table 4:** Mean differences and ranking of exercise motivations between U.S. and Korean male college students.

Subscale	Nationality	*N*	*M*	*SD*	Rank	*Z*	*p*	Cohen’s *d*
Stress management	U.S.	184	4.24	1.29	8	−4.149	<0.001	0.39
	Korea	185	3.80	0.95	11			
Revitalization	U.S.	184	4.57	1.05	3	−2.376	0.017	0.21
	Korea	185	4.36	0.98	4			
Enjoyment	U.S.	184	4.39	1.27	6	−0.357	0.721	−0.06
	Korea	185	4.46	0.94	3			
Challenge	U.S.	184	3.89	1.39	10	−2.218	0.027	−0.37
	Korea	185	4.31	0.81	5			
Social recognition	U.S.	184	3.19	1.54	13	−3.688	<0.001	−0.45
	Korea	185	3.77	0.96	13			
Affiliation	U.S.	184	3.38	1.48	12	−2.760	0.006	−0.32
	Korea	185	3.80	1.10	10			
Competition	U.S.	184	3.71	1.48	11	−0.210	0.833	−0.07
	Korea	185	3.80	1.18	12			
Health pressures	U.S.	184	2.87	1.37	14	−2.324	0.020	−0.19
	Korea	185	3.11	1.08	14			
Ill-health avoidance	U.S.	184	4.49	1.12	4	−2.80	0.005	0.26
	Korea	185	4.22	0.96	8			
Positive health	U.S.	184	5.09	0.85	1	−5.691	<0.001	0.53
	Korea	185	4.67	0.74	2			
Weight management	U.S.	184	4.08	1.25	9	−0.584	0.559	0.07
	Korea	185	4.00	1.22	9			
Appearance	U.S.	184	4.44	1.02	5	−2.102	0.036	0.19
	Korea	185	4.25	0.94	7			
Strength and endurance	U.S.	184	4.86	1.08	2	−2.036	0.042	0.09
	Korea	185	4.78	0.76	1			
Nimbleness	U.S.	184	4.33	1.26	7	−0.659	0.510	0.04
	Korea	185	4.29	1.04	6			

A comparison of exercise motivation rankings for U.S. and Korean male college students indicated that positive health was the highest ranked exercise motivation for U.S. male college students, while Korean male students ranked strength/endurance most highly. Both groups of male students ranked health pressures lowest among all motivations for exercise. In addition, enjoyment and challenge were clearly stronger motivations for exercise for Korean male college students, while stress management and ill-health avoidance were more important for U.S. male college students.

## 4 Discussion

This study revealed distinct motivational profiles among U.S. and Korean college students and showed that several dimensions of exercise motivation differed across the two student populations. These findings do more than document between-group differences; they suggest that the relative importance of specific exercise motives may vary across educational and social contexts. In particular, the results indicate that exercise motivation among college students is not organized in exactly the same way across national settings, which has implications for both theory and intervention design. The findings offered partial support for the proposed hypotheses, with statistically significant group differences observed on several exercise motivation subscales rather than across the full set of motives. Taken together, the findings are broadly consistent with Self-Determination Theory in suggesting that exercise motivation is not a single undifferentiated construct, but rather a pattern of motives that may vary in salience across settings and subgroups. Recent research has continued to support the usefulness of Self-Determination Theory for understanding exercise motivation among university students, especially by showing that different motives can be associated with physical activity engagement and mental health outcomes (13, 14). At the same time, the present study was not designed to directly test the social or psychological mechanisms underlying these differences; therefore, the discussion focuses on theoretically informed interpretations rather than causal explanations.

Across both Korean and U.S. college students, positive health and strength/endurance emerged as the most prominent exercise motives. This shared pattern suggests that health maintenance and physical capability remain central reasons for exercise participation regardless of cultural context (34–36). At the same time, the groups differed in the relative importance assigned to other motives, indicating that cultural context shapes how students prioritize specific reasons for exercise. The study therefore contributes by suggesting that exercise motivation among college students may involve both shared health-oriented motives and motives whose salience varies across contexts. Because cultural values were not directly measured, these differences should be interpreted cautiously. Interestingly, U.S. students ranked ill-health avoidance as their third most frequent exercise motivation, whereas Korean students prioritized enjoyment. From an SDT perspective, this pattern may indicate that different motives become more salient across settings, with some students placing greater emphasis on personally valued health management and others on the enjoyment or experiential quality of exercise participation (13, 16). However, because the present study did not directly assess broader value orientations, peer norms, or institutional influences, these interpretations should be regarded as tentative rather than definitive. U.S. students may have been more likely to view exercise as a purposeful strategy for preventing health problems and supporting personal well-being, which is consistent with prior research highlighting individual agency and self-care in the U.S. context (20). By contrast, Korean students may have placed relatively greater value on enjoyment because exercise participation in collectivist settings can be intertwined with social interaction and recreational engagement (18). These interpretations may be consistent with earlier studies reporting cultural variation in exercise motivation, particularly differences between health-centered motives in Western contexts and socially oriented motives in collectivist contexts such as South Korea (13, 17). However, because cultural values were not directly measured in the present study, this interpretation should be regarded as tentative. More broadly, the findings may be consistent with prior work indicating that contextual factors can influence which exercise motives become more salient in different settings (37). However, because the present study did not directly assess cultural orientations or related mechanisms, these differences should be interpreted as preliminary evidence rather than as direct support for specific cultural explanations. An examination of effect sizes indicated that several group differences were meaningful in magnitude. In particular, stress management showed the most pronounced difference, suggesting that the use of exercise as a strategy for coping with psychological stress varies substantially across cultural contexts. Positive health and ill-health avoidance also reflected moderate differences, indicating that health-related motivations tend to be more salient among U.S. students. In contrast, the difference in social recognition was relatively small, implying that although Korean students reported higher levels on this motive, the overall gap between groups was modest.

Korean students placed higher importance on social recognition, particularly among males. One possible interpretation is that exercise participation in this group may be more closely connected to interpersonal visibility, peer-related meaning, or group-oriented forms of participation. However, because these underlying processes were not directly measured in the present study, the interpretation remains tentative. Recent evidence suggests that peer relationships and social support can play a meaningful role in shaping exercise-related attitudes and intentions among young people and college students (18, 26). By contrast, U.S. students demonstrated stronger motivations for stress management and self-improvement. This pattern may reflect a stronger tendency to view exercise as a personally valued strategy for health management and psychological well-being, although the present study does not allow direct conclusions about broader social values (38). In the secondary subgroup analyses, gender-related patterns emerged as additional descriptive findings, although gender was not modeled as a principal explanatory factor in the present study: U.S. female students consistently scored higher than Korean female students across all EMI-2 subscales, indicating broader and higher internalization of exercise motivation. These findings echo global surveys showing persistent gender disparities in physical activity, where women in East Asian countries report lower activity levels due to social barriers, modesty norms, and limited access to structured programs (23, 39). Previous studies indicate that in Korea, male children or youths are more often involved in physical activity, sports, and exercise than females (40, 41). This tendency may continue in college students’ exercise participation trends, with males being more active in leisure time than females (37). These patterns may be consistent with the possibility that gendered participation norms influence exercise motivation in this context, although such norms were not directly measured in the present study. Korean women may encounter implicit cultural barriers, such as modesty norms or limited exposure to structured physical activity during adolescence (23, 41, 42). The differences observed among female students were noteworthy within the secondary subgroup analyses, but they should be interpreted as supplementary to the primary cross-cultural comparison. In particular, stress management showed a very large effect, while positive health, appearance, and strength/endurance were associated with moderate-to-large effects. Taken together, these patterns indicate that the group differences among female students are meaningful from a practical standpoint. In contrast, the higher motivation observed in U.S. female students may be attributed to greater emphasis on fitness culture in Western societies, where gym participation and sports engagement are actively encouraged from an early age (38, 43), and where fitness culture has been actively promoted, especially among women, for both health and body image reasons (18, 20).

Results of exercise motivation preferences indicated both U.S. and Korean male college students chose “positive health” and “strength and endurance” as their top two motivations, supported by previous studies (19, 24). When comparing exercise motivations of U.S. and Korean male college students, U.S. males exhibited higher motivation for stress management, revitalization, and physical health, whereas Korean males demonstrated greater motivation for challenge, social recognition, and affiliation. These findings are broadly consistent with Self-Determination Theory, in that stress management, revitalization, and physical health may reflect more self-regulated or internally endorsed motives, whereas social recognition and affiliation may reflect more externally or socially oriented motives. Recent research has continued to support the usefulness of Self-Determination Theory for understanding exercise motivation among university students, especially by showing that different motives can be associated with physical activity engagement and mental health outcomes (13, 14). Accordingly, the results suggest variation in motivational orientation across contexts, although the present study does not support a strict cultural dichotomy (10, 20, 44). In contrast to the female sample, most effect sizes among male students were small to moderate. The largest differences were observed for social recognition, positive health, and challenge, indicating moderate practical differences. Other significant findings, such as appearance and strength/endurance, reflected relatively small effects despite reaching statistical significance.

The present findings may also help clarify how cultural and institutional contexts shape the relative salience of exercise motives. Although cultural values were not directly measured in this study, the observed pattern may be consistent with the view that social and educational environments influence which motives become more prominent in student populations. This interpretation is tentative, but it suggests that exercise motivation is not only a function of individual preference; it is also shaped by the settings in which students live, study, and exercise. From a practical perspective, these findings may inform physical activity promotion strategies by indicating that interventions should be aligned with the motives that are most meaningful in each context. Programs for Korean students may benefit from greater emphasis on social recognition, peer involvement, and group-based participation, whereas programs for U.S. students may be more effective when they highlight stress management, health maintenance, and personal well-being.

### 4.1 Limitations

Several limitations should be considered when interpreting the findings of this study. The main limitation is that other factors, such as circumstances or environment, might also be associated with differences in college students’ exercise motivations. For instance, Korean colleges offer limited access to recreation centers or sport facilities on campus, while most U.S. colleges provide various sports, wellness programs, and recreation centers to encourage students to exercise. Another example of different circumstances is that U.S. college students are familiar with exercising because many of them have experience in youth sport teams or individual sports, but few Korean college students participate in organized sports during middle or high school. Another primary limitation is the restricted generalizability of the findings, as the study used convenience sampling and included participants from only a small number of universities in specific regions of the United States and South Korea. Additionally, the ability to generalize from this study is limited due to the convenience sampling, which might generate bias in gathering data or create sampling errors. The use of self-report measures in this study warrants caution, as responses may be influenced by common method variance and tendencies toward socially desirable answering. To address these concerns, future studies could benefit from incorporating alternative data sources or combining multiple methods, along with design-based strategies aimed at minimizing such biases. Finally, this study does not address personal characteristics such as race, socioeconomic status, or health capabilities. These characteristics might affect students’ motivation to exercise. Future research should address these limitations and further examine exercise motivation among U.S. and Korean college students.

### 4.2 Implications for future research and practice

These insights provide valuable implications for the development of culturally sensitive exercise programs. Interventions for Korean students may be more effective when they incorporate social recognition and group-based participation, consistent with the stronger role of social recognition in this sample. In contrast, interventions for U.S. students may benefit from emphasizing stress management and psychological well-being, reflecting the higher stress management motivation observed among U.S. participants. Conversely, personalized fitness plans in the U.S. may align better with students’ intrinsic motivations (19, 23). In addition, the comparatively lower motivation of Korean female students indicates the importance of creating supportive and accessible exercise settings that can reduce participation barriers (23, 35).

Future research should explore additional variables, such as socioeconomic status, psychological factors, and access to sports facilities, to further elucidate exercise motivation across diverse demographics. Longitudinal studies may also provide a deeper understanding of how exercise motivations evolve throughout college life (19, 20). By recognizing the cultural influences on exercise motivation, policymakers and educators can develop more effective strategies to promote physical activity among college students globally. Overall, the findings point to a critical implication: exercise motivation is not a universal construct. Cultural and gendered contexts must be considered when developing interventions or public health initiatives to promote physical activity. Programs in Korea might benefit from emphasizing social aspects of exercise, such as team-based activities or peer-led fitness challenges. Meanwhile, U.S. programs could continue to support individual health goals but should remain attentive to students who may not resonate with the dominant fitness culture.

## 5 Conclusion

This study compared exercise motivation between U.S. and Korean college students using the Exercise Motivations Inventory-2 (EMI-2) and identified meaningful differences in motivational profiles across the two student populations. Across groups, positive health and strength/endurance emerged as the most highly endorsed motives, suggesting that some health-oriented reasons for exercise remain prominent across settings. At the same time, motives such as stress management, enjoyment, and social recognition differed in their relative salience, indicating that exercise motivation among college students is not organized in exactly the same way across national contexts.

Beyond documenting group differences, the findings contribute to exercise motivation research by showing that some motives appear relatively stable across student populations, whereas others may be more sensitive to the educational and social environments in which students are embedded. In this sense, the comparison between the United States and South Korea is informative not simply because the two countries differ, but because it helps clarify how exercise motives may vary across distinct participation contexts. Although cultural values and related mechanisms were not directly measured in the present study, the results are consistent with the view that exercise motivation is shaped not only by individual preference but also by broader contextual conditions. The secondary subgroup analyses further suggested that motivational patterns may vary within gender groups, although the primary contribution of the study remains the cross-national comparison. Taken together, these findings may inform the development of context-sensitive strategies for promoting physical activity in university settings, including approaches that place greater emphasis on social engagement in some contexts and stress management or personal well-being in others.

## Data Availability

The original contributions presented in the study are included in the article/supplementary material, further inquiries can be directed to the corresponding author.
